# Creatine kinase elevation after robotic surgery for rectal cancer due to a prolonged lithotomy position

**DOI:** 10.1186/s12893-020-00771-2

**Published:** 2020-06-16

**Authors:** Yuki Tsuchiya, Shinya Munakata, Ryoichi Tsukamoto, Yu Okazawa, Kosuke Mizukoshi, Kiichi Sugimoto, Makoto Takahashi, Yutaka Kojima Yuichi Tomiki, Kazuhiro Sakamoto

**Affiliations:** Department of Coloproctological Surgery, Juntendo University Faculty of Medicine, 2-1-1 Hongo, Bunkyo-ku, Tokyo, 113-8421 Japan

**Keywords:** Rhabdomyolysis, Creatine kinase, Conventional laparoscopic surgery, And robotic-assisted laparoscopic surgery

## Abstract

**Background:**

Robotic surgery for rectal cancer, which is now performed worldwide, can be associated with elevated creatine kinase levels postoperatively. In this study, we compared postoperative complications between patients undergoing robotic surgery and laparoscopic surgery.

**Methods:**

We identified 66 consecutive patients who underwent curative resection for rectal cancer at Juntendo University Hospital between January 2016 and February 2019. Patients were divided into a conventional laparoscopic surgery (CLS) group (*n* = 38) and a robotic-assisted laparoscopic surgery (RALS) group (*n* = 28) before comparing various clinicodemographic factors between the groups.

**Results:**

Patient age and gender, surgical approach (CLS/RALS), pathological T factor, pathological stage, duration of postoperative hospital stay, and postoperative complications were not significantly different between the RALS and CLS groups. However, the operation time was significantly longer in the RALS group (407 min) than in the CLS group (295 min; *p* < 0.001). Notably, the serum level of creatine kinase on postoperative day 1 was significantly higher in the CLS group (154 IU/L) than in the RALS group (525 IU/L; *p* < 0.001), despite there being no significant differences in the incidence of rhabdomyolysis. The multivariate analysis showed that RALS/CLS (HR 6.0 95% CI 1.3–27.5, *p* = 0.02) and operation time (HR 15.9 95% CI 3.79–67.4, *p* = 0.001) remained independent factors of CK elevation on postoperative day 1.

**Conclusions:**

Clinically relevant positioning injuries and rhabdomyolysis may occur in patients who are subjected to a prolonged and extreme Trendelenburg position or who have extra force applied to the abdominal wall because of remote center displacement. The creatine kinase value should therefore be measured after RALS to monitor for the sequelae of these potential positioning injuries.

## Background

Robotic-assisted laparoscopic surgery (RALS) in the lithotomy position is often used in urological, gynecological, and colorectal surgery, but the potentially devastating creatine kinase (CK)-related complications, such as rhabdomyolysis and acute compartment syndrome (ACS), are not widely recognized. Serum CK increases after surgery, peaking at 18 h after the procedure, but serum CK elevation should not be used to predict positioning injury [1]. In rectal cancer, laparoscopic surgery has a higher conversion rate and a higher positivity rate for circumferential resection margins compared with open surgery [2]. However, RALS using the da Vinci Surgical System (Intuitive Surgical, Sunnyvale, CA) benefits from free-moving multi-joint forceps, high-quality three-dimensional imaging, stable camera work, image stabilization, motion-scaling, and greatly improved ergonomics. Thoughtful positioning can minimize the risk of iatrogenic injury, including peripheral nerve damage, rhabdomyolysis and ACS, by ensuring adequate access.

ACS is uncommon in patients undergoing prolonged colorectal procedures, but when it develops, it can have catastrophic consequences if metabolic acidosis and myoglobinuric renal failure develop. The estimated incidence of ACS is 1 in 3500 among patients who undergo procedures in the lithotomy position [3]. ACS can also result in rhabdomyolysis, and in doing so, can cause acute renal failure, which is signaled by the onset of hyperkalemia, hyperphosphatemia, hypocalcemia, and metabolic acidosis. The precise mechanisms of acute renal failure in rhabdomyolysis are unclear, but most studies point to tubule obstruction by myoglobin precipitation, tubular necrosis, lipid peroxidation, and renal vasoconstriction due to hypovolemia and vasoactive medication use. The cornerstone of prevention for acute renal failure is aggressive and early volume replacement [4], although the creatine kinase (CK) level will also determine the degree of muscle necrosis and rhabdomyolysis. Currently, little is known about how frequently rhabdomyolysis occurs or additional complications due to the operation time, lithotomy position, and extra force required when using the da Vinci system. In this study, we therefore compared postoperative complications between patients undergoing RALS and conventional laparoscopic surgery (CLS) by monitoring serum CK.

## Methods

### Study design and participants

We reviewed the cases of 66 consecutive patients who underwent curative resection of rectal cancer at Juntendo University Hospital between January 2016 and February 2019.This was a retrospective study and was approved by our hospital’s Institutional Review Board, which waived the requirement for patient consent. The following cases were excluded from analysis: emergencies, double cancers, and stage IV cancers. Operation techniques such as lateral lymph node resection, abdominoperineal resections, and intersphincteric resections were also excluded because of extensive levator ani muscle damage. Operative procedures were decided through preoperative meetings.

CLS was performed in 38 patients and RALS was performed in 28 patients (Si = 20, Xi = 8). We divided the patients into a CLS group and a RALS group before comparing the following factors between those groups: age, gender, body mass index, tumor location, surgical approach (CLS or RALS), surgical procedure, distance of the tumor from the margin of the anus, T factor, stage, postoperative serum CK level, duration of postoperative hospital stay, and postoperative complications.

### Operative techniques

#### Robotic procedure

We used the da Vinci Surgical System (Intuitive Surgical, Sunnyvale, CA, USA) for the robotic procedure. This system consists of a surgeon’s console, an electronic tower that holds the video equipment, and robotic arms. Patients were placed supine and with their legs apart in a 15°–20° Trendelenburg and 8°–10° right-down position. The robot was docked off the patient’s left thigh. A 12 mm trocar was used by inserting da Vinci 8 mm port. The left colon was mobilized after dividing the inferior mesenteric artery and vein using clips, and the robotic instrument was sited between the patients’ legs. The rectum was suspended from the assistant’s port, using gauze, and we proceeded according to the principles of total mesorectal excision.

The mesorectum was precisely divided beyond the tumor using both robotic arms. The tumor location was checked by colonoscopy. The rectum was divided using an endoscopic linear stapler or da Vinci stapler, the robotic instrument was disengaged, and the specimen was extracted though the lower umbilical trocar incision, which was enlarged to approximately 4 cm. An anvil head was then inserted in the proximal colon and secured with a purse-string suture. The pneumoperitoneum was restored before a circular stapler was used to create an end-to-end anastomosis. Before this initiating study, the surgeons undertook more than 10 da Vinci surgeries because the learning curve for RALS procedures is achieved after 15–25 cases [5]. Patients were enrolled in this study only after surgical procedures were standardized.

#### Laparoscopic procedure

A 12-mm trocar was inserted at the umbilicus for a 30° standard laparoscope to be inserted. Another three 5 mm trocars were used in the left lower quadrant, and bilaterally in the mid-abdomen (adjacent the umbilicus) along the midaxillary line. A 12 mm trocar was added to the right lower quadrant to facilitate use of an endoscopic linear stapler. Left colon mobilization was performed in a medial to lateral fashion. The inferior mesenteric artery was divided at its root by an endo-clip, and rectal dissection in the mesorectal plane proceeded using conventional laparoscopic instruments for total mesorectal excision. The mesorectum was divided precisely beyond the tumor with an ultrasonic device, and the rectum itself was divided with an endoscopic linear stapler. Specimen extraction and reconstruction were the same as in the robotic procedure.

#### Other procedures

Rectal anastomoses were performed with a double stapling technique. Patients operated on by the authors were given the option of CLS or RALS although the final decision was at the surgeon’s discretion. Temporary ileostomies were also created selectively at the surgeon’s discretion, based on consideration of whether the anastomosis site was below 5 cm from the anal verge, whether the patient used steroids or had diabetes, and whether they had received preoperative neoadjuvant chemotherapy.

### Definitions

Each barium enema for tumor assessment was retrospectively reviewed for rectal cancer. The rectum was divided into upper and lower regions. When the tumor was located between the inferior margin of the second sacral vertebra and the peritoneal reflection, its location was recorded as the upper rectum. When the tumor was located below the peritoneal reflection, its location was recorded as the lower rectum. CT and MRI were performed for preoperative diagnosis in all cases. Finally, preoperative neoadjuvant chemotherapy (e.g., FOLFOX or CAPOX) was performed for rectal cancer graded as cT3, cT4a, cT4b, any c N+, and cM0 according to the TNM Classification of Malignant Tumors, seventh edition. The staging of all cancers was according to the TNM classification.

Anastomotic leakage was defined clinically by the presence of a pelvic abscess, fecal discharge from the wound and drain, septicemia, and peritonitis, with or without radiologically confirmed leakage [6]. Postoperative ileus was defined as an inability to tolerate food in the presence of abdominal distention, absent bowel sounds, and a need to delay enteral feeding [7]. Blood tests were performed every other day after the operation. CK normal levels ranged from 47 to 240 U/L in our hospital. A high CK level was defined as that over 240 U/L. Rhabdomyolysis was defined by serum CK levels > 5000 IU/L [8]. ACS was defined as high pressure within a closed fascial space (muscle compartment) causing reduced capillary blood perfusion below the level necessary for tissue viability [9]. Calf compressors were used in all cases to prevent deep vein thrombosis.

These postoperative complications were stratified by the Clavien–Dindo classification [10]. Finally, operative mortality was compared based on a definition as any death that occurred within 30 days after the primary operation.

### Statistical analysis

We used JMP version 10 software (SAS Institute Inc., Cary, N.C., USA) for the statistical analysis. Categorical variables were compared using chi-squared or Fisher’s exact tests, as appropriate. Continuous variables are presented as medians and were compared using the Mann–Whitney *U* test or analysis of variance. The Spearman’s rank correlation coefficient was used to evaluate correlations. For multivariate analysis, the Cox proportional-hazard regression model was used with the hazard ratio (HR).

## Results

### Patient characteristics

The study comprised 38 males and 28 females with ages ranging from 37 to 92 years (median 67 years). The most common tumor location was the upper rectum (49 patients; 74%), with less identified in the lower rectum (17 patients; 26%). Cancers were classified pathologically as T1, T2, T3, and T4 in 16 (24%), 15 (23%), 31 (47%), and 4 (6%) patients, respectively. In addition, 22 (33%) had stage I disease, 17 (26%) had stage II disease, and 27 (41%) had stage III disease. The median tumor distance from the anal verge was 8.5 cm. Ileostomy to rest the anastomosis was required for 37 patients (56%). Neoadjuvant chemotherapy with FOLFOX and CAPOX was given to 5 patients and 1 patient, respectively. As for complications, anastomotic leakage occurred in 1 patient (Clavien–Dindo complication grade II) and 2 patients (grade IIIa).

Three patients developed postoperative ileus (grade II), 2 patients developed rhabdomyolysis (grade II), and 1 patient developed ACS (grade II). The median number of postoperative days in hospital was 12 (range, 8–73 days).

### Comparison of the CLS and RALS groups

The short-term outcomes are shown in Table 1. Notably, age, gender, tumor location, approach (CLS or RALS), pathological T factor, pathological stage, postoperative hospital stay, and postoperative complications were comparable between the RALS and CLS groups. The activities of most serum skeletal muscle enzymes, such as alanine aminotransferase, aspartate aminotransferase, and lactate dehydrogenase, were not significantly different between the RALS and CLS groups (data not shown).

**Table 1:** Characteristics and outcomes of patients undergoing conventional and robotic-assisted laparoscopic surgery

	CLS (*N* = 38)	RALS (*N* = 28)	*P*-value
Sex(M/F)	21/17	17/11	0.65
Age (years)	69 (37-92)	65 (37-80)	0.22
BMI (kg/M^2^)	21.6 (16-29.3)	22.4 (16.4-31.4)	0.29
Tumor location (upper/lower rectum)	24/14	25/3	0.01
Neoadjuvant chemotherapy	6	0	0.03
Operation time (min)	295 (193-545)	407 (276-651)	0.001
Blood lost (g)	21 (5-90)	25 (10-80)	0.31
Ileostomy	17	20	0.04
Complications			
Anastomotic leakage	3	0	0.06
Ileus	2	1	0.81
Rhabdomyolysis	1	1	0.67
Acute compartment syndrome	1	0	0.29
Reoperation	2	0	0.22
Post operative day (day)	11.5 (8-73)	12 (8-28)	0.82
pathological T categories(1/2/3/4)	7/10/17/4	9/5/14/0	0.09
pstage (I / II / III)	12/10/16	10/7/11	0.33

Abbreviations: *BMI* body mass index, *CLS* conventional laparoscopic surgery, *RALS* robotic-assisted laparoscopic surgery

**Fig. 1:**
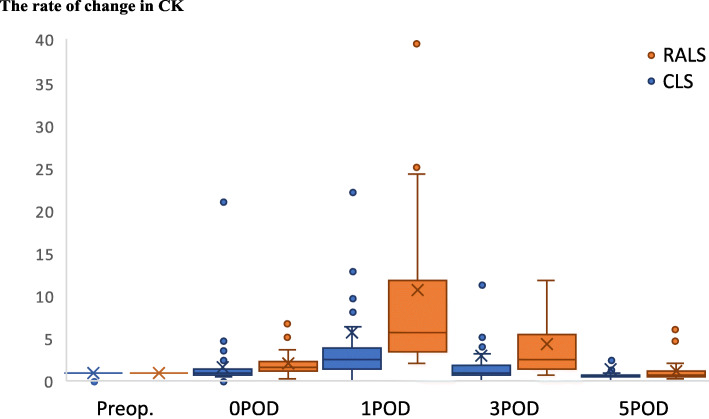
Relationship between conventional and robotic-assisted laparoscopic approaches on serum creatine kinase levels by postoperative day. Bars represent mean ± standard deviation. Abbreviations: CK, creatine kinase; CLS, conventional laparoscopic surgery; POD, postoperative day; RALS, robotic-assisted laparoscopic surgery

**Fig. 2:**
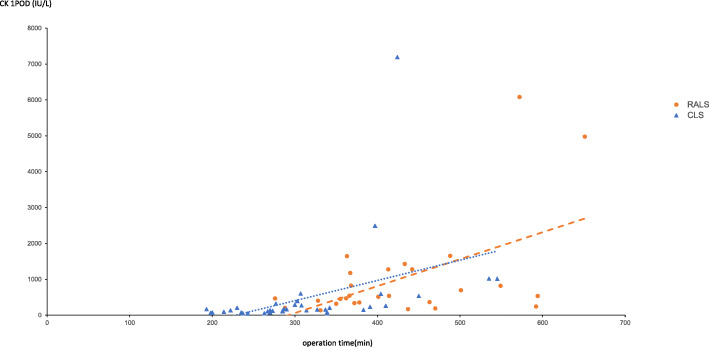
Relationship between operation time and serum creatine kinase on postoperative day 1. Abbreviations: POD1, postoperative day 1; CK, creatine kinase; CLS, conventional laparoscopic surgery; RALS, robotic-assisted laparoscopic surgery

We did find that the operative time was significantly longer in the RALS group (407 min) than in the CLS group (295 min; *p* < 0.001). In addition, serum CK levels on postoperative day 1 were significantly higher in the RALS group (525 IU/L) compared with the CLS group (154 IU/L; *p* < 0.001). The differences in serum CK levels also remained significantly higher in the RALS group on postoperative days 3 (p < 0.001) and 5 (*p* = 0.03) (Fig. 1). Of note, the serum CK level tended to increase with operation time in both the CLS group (r = 0.40, *p* < 0.0001) and the RALS group (r = 0.53, *p* = 0.03) (Fig. 2), indicating that CK levels may be related to time spent in the lithotomy position and to pressure applied to the abdominal wall.

Rhabdomyolysis occurred in one patient per group, and one other patient in the CLS group had severe leg pain after the operation (diagnosed as ACS). However, these cases showed improvement with fluid infusions. There were no cases of acute renal failure associated with rhabdomyolysis or elevated CK levels. There were no significant differences between the CLS and RALS groups in the rates of postoperative ileus (2 patients and 1 patient, respectively; *p* = 0.81) or anastomotic leakage (3 patients in the CLS group only; *p* = 0.81). As for reoperation, ileostomy was required to manage anastomotic leakage in 2 patients in the CLS group. There were no conversions in either group. Finally, there were no operative mortalities.

### Etiology of CK elevation

Patients were divided into low and high subgroups of CK elevation based on our hospital’s normal range. The results are shown in Table 2. We determined that the operative time was significantly longer in the high CK group (407 min) than in the low CK group (286 min) (*p* < 0.001). In addition, surgical procedures differed significantly between the two groups. In the low CK group, 28 patients (87.5%) were operated using CLS, and 4 patients (12.5%) were operated using RALS. On the other hand, in the high CK group, 10 patients (29.4%) were operated using CLS, and in 24 cases (70.6% were operated using RALS (*p* < 0.001). Multivariate analysis showed that CLS/RALS (HR = 6.0, 95% CI 1.3–27.5, *p* = 0.02) and operation time (HR = 15.9, 95% CI 3.79–67.4, *p* = 0.001) remained independent factors of CK elevation.

**Table 2:** Univariate and multivariate analysis of factors in CK elevation

	CK Low (*N* = 32)	CK High (*N* = 34)	Univariate P	Multivariate P	HR	95% CI
BMI (kg/M^2^)	21.8 (16.5-29.3)	22.3 (16-31.4)	0.96			
Tumor location (upper/lower rectum)	23/9	26/8	0.67			
Neoadjuvant chemotherapy	4	2	0.35			
CLS/RALS	28/4	10/24	0.001	0.02	6	1.3-2.5
Operation time (min)	286 (193-470)	407 (276-651)	0.001	0.001	15.9	3.79-67.4
Blood lost (g)	23 (5-90)	23 (8-110)	0.75			
Ileostomy	16	21	0.34			
pstage (I / II / III)	10/8/14	12/9/13	0.89			

*CI* Confidence interval, *HR* Hazard Ratio

## Discussion

Compared to laparoscopic rectal cancer surgery, RALS has benefits of providing an immersive 3-dimensional depth of field, articulating instruments, and a stable camera platform. It has been introduced in many facilities despite its cost, including the capital and ongoing maintenance charges [11]. Furthermore, sexual function may recover better with RALS than with CLS [12]. We showed that there was an increased risk of CK elevation during RALS, presumably due to both prolonged stasis in the lithotomy position and positioning injury. Although ACS occurred in one patient in the CLS group, serum CK levels were higher in the RALS group, indicating a higher risk of rhabdomyolysis. The operation time appears key to such injuries. For example, Sajid et al. reviewed 34 cases of ACS associated with colorectal surgery in the lithotomy position (16 unilateral and 13 bilateral cases) and reported that the mean operation time was 435 min, with most cases diagnosed within 24 h of surgery [13].

The risk factors for ACS are reported to be prolonged surgery, obesity, head-down tilt position, and elastic stocking use. Laparoscopic surgery further disturbs venous return due to pressure from the pneumoperitoneum [14]. In addition, the robots used in RALS lack the sense of touch (i.e., haptics) and can apply excessive pressure on the abdominal wall or legs. During surgery, the sense of touch gives immediate feedback about what is being done, the degree of tissue tension, and the force applied when manipulating a suture. By contrast, robotic arms that lack haptic feedback are more prone to accidental pressure-induced trauma in the prolonged lithotomy position. While docking the robot, to minimize risk when setting its arms, the surgeon should also be aware of the potential for trauma.

**Fig. 3:**
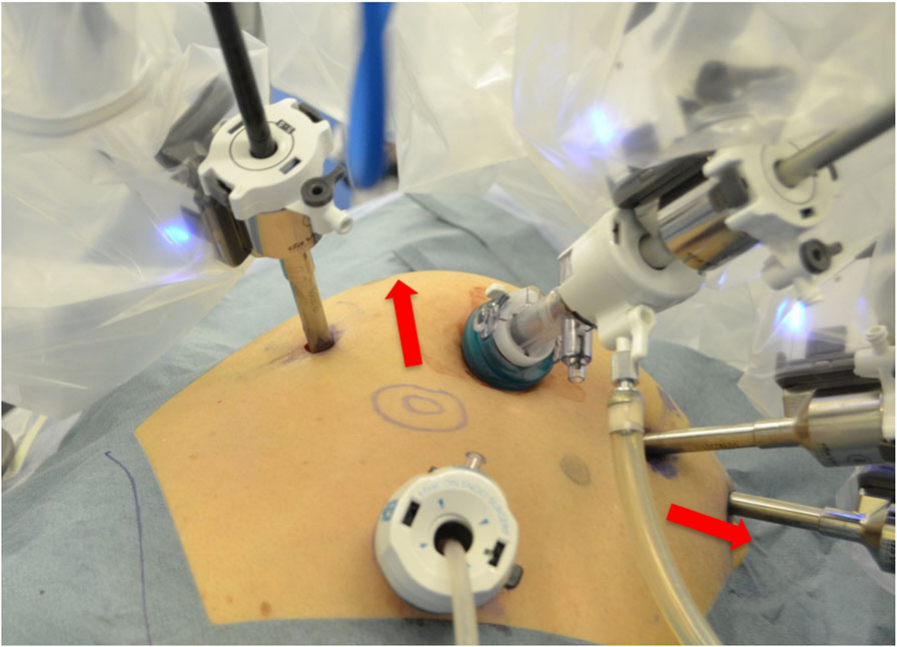
Pressure to the abdominal wall caused by the da Vinci port. The arrows show the pressure site on the abdominal wall

We showed that RALS remained an independent factor for CK elevation. In general, the da Vinci port moves with the remote center as its fulcrum, and although this should adjust to the abdominal wall, deviation can occur. If the remote center is displaced, then the abdominal wall can be pulled by the trocars, as we have experienced in practice (Fig. 3). Therefore, assistants should check the position of the remote center, paying attention to any traction on the abdominal wall. After docking, we advocate pushing the port clutch button to release any pressure applied to the abdominal wall. Although the size of robotic arms have improved in new Xi systems, CK levels have not changed significantly from the Si systems in our experience (data not shown). In this study, elevations in serum CK levels were observed without clinical ACS or rhabdomyolysis, except when the lithotomy position was inadequate. All cases that developed CK elevations gradually improved with fluid infusions.

Yamaguchi et al. reported that RALS was associated with less blood loss, shorter postoperative hospital stays, and lower rates of urinary retention, but no significant benefit in frequencies of anastomotic leakage or small bowel obstruction [15, 16, 4]. Although there have been few reports of complications directly related to the robot itself, those that have occurred were related to the peripheral nervous system and to the cardiac and ophthalmic systems [17]. Mattei et al. suggested that positioning injuries were most common in the gluteal area and recommended special care by ensuring adequate gluteal cushioning when positioning [1]. Risk factors for these injuries were a high body mass index, a long operative time, and a long time in the Trendelenburg position [1, 18]. We showed that operation time also remained an independent factor of CK elevation, but this study did not show any difference in BMI. We also note that time in the Trendelenburg position can lead to positioning injuries. Recently, we therefore introduced a repositioning protocol in which the patient must be returned to a supine position for 10 min every 3 h. Some authors have also suggested repositioning the legs during excessively long operations [19]. Given that elevation in CK occurs, however, the importance of monitoring serum CK should not be underestimated if we are to prevent rhabdomyolysis.

## Conculsion

Postoperative CK measurement is important to the identification and monitoring of postoperative rhabdomyolysis. Unfortunately, the results of the current study are limited by its retrospective design and small sample size, so a validation study with multivariate analysis that includes all relevant factors will be needed. However, it should be noted that this study included early case experience using the da Vinci system in our hospital, and with time, we anticipate that surgical learning curves will shorten and that experience will improve our practice further. In the meantime, we advocate that all surgical staff should pay close attention to the patient’s position and to any pressure on the abdominal wall during RALS.

## Data Availability

The datasets used and analysed during the current study are available from the corresponding author on reasonable request.

## Abbreviations

ACS: Acute compartment syndrome
CI: Confidence interval
CK: creatine kinase
CLS: Conventional laparoscopic surgery
HR: Hazard Ratio
RALS: Robotic-assisted laparoscopic surgery
